# Sialylation and fucosylation modulate inflammasome-activating eIF2 Signaling and microbial translocation during HIV infection

**DOI:** 10.1038/s41385-020-0279-5

**Published:** 2020-03-09

**Authors:** Leila B. Giron, Ceylan E. Tanes, Mariane H. Schleimann, Phillip A. Engen, Lisa M. Mattei, Alitzel Anzurez, Mohammad Damra, Huanjia Zhang, Kyle Bittinger, Frederic Bushman, Andrew Kossenkov, Paul W. Denton, Hiroaki Tateno, Ali Keshavarzian, Alan L. Landay, Mohamed Abdel-Mohsen

**Affiliations:** 1 The Wistar Institute, Philadelphia, PA USA; 2 Division of Gastroenterology, Hepatology, and Nutrition, Children’s Hospital of Philadelphia, Philadelphia, PA USA; 3 Department of Infectious Diseases, Aarhus University Hospital, Aarhus, Denmark; 4 Division of Digestive Diseases and Nutrition, Department of Internal Medicine, Rush University Medical Center, Chicago, IL USA; 5 University of Pennsylvania, Philadelphia, PA USA; 6 University of Nebraska Omaha, Omaha, NE USA; 7 National Institute of Advanced Industrial Science and Technology (AIST), Tsukuba, Japan; 8 Division of Geriatric, Department of Internal Medicine, Rush University Medical Center, Chicago, IL USA

## Abstract

An emerging paradigm suggests that gut glycosylation is a key force in maintaining the homeostatic relationship between the gut and its microbiota. Nevertheless, it is unclear how gut glycosylation contributes to the HIV-associated microbial translocation and inflammation that persist despite viral suppression and contribute to the development of several comorbidities. We examined terminal ileum, right colon, and sigmoid colon biopsies from HIV-infected virally-suppressed individuals and found that gut glycomic patterns are associated with distinct microbial compositions and differential levels of chronic inflammation and HIV persistence. In particular, high levels of the pro-inflammatory hypo-sialylated T-antigen glycans and low levels of the anti-inflammatory fucosylated glycans were associated with higher abundance of glycan-degrading microbial species (in particular, *Bacteroides vulgatus)*, a less diverse microbiome, higher levels of inflammation, and higher levels of ileum-associated HIV DNA. These findings are linked to the activation of the inflammasome-mediating eIF2 signaling pathway. Our study thus provides the first proof-of-concept evidence that a previously unappreciated factor, gut glycosylation, is a force that may impact the vicious cycle between HIV infection, microbial translocation, and chronic inflammation.

## Introduction

HIV infection causes changes in gut structure and a breakdown of the epithelial barrier, which can increase permeability to gut microbes and microbial products.^1^ This microbial translocation is thought to be a major cause of local and systemic immune activation and inflammation that may further increase or sustain HIV replication, resulting in a positive feedback cycle.^1–4^ Immune activation and inflammation contribute to the development of non-AIDS comorbidities such as cardiovascular diseases and neurological impairments,^1,5,6^ and possibly viral persistence.^2,4^ Unfortunately, even with antiretroviral therapy (ART), the damage to the epithelial barrier caused by HIV is not fully repaired, allowing microbial translocation and inflammation and their clinical sequalae to continue.^7^ Several studies have shown that this HIV-associated microbial translocation may be associated with changes in the composition and diversity of the enteric microbiome;^8–10^ These changes are multifactorial in origin,^11–13^ resulting from both sexual behaviors and effects of HIV infection itself.^14^ A comprehensive understanding of the host factors that drive microbial translocation and gut inflammation during HIV infection is essential for developing strategies to treat them. Here, we start to investigate whether an unappreciated factor—gut glycosylation—plays a role in the positive feedback cycle between HIV, microbial translocation, and chronic inflammation during ART-suppressed HIV infection.

An emerging paradigm suggests that the gut glycome is critical for maintaining a homeostatic relationship between the host and its gut microbiota. Gut epithelial cells and the gut mucus layer are heavily glycosylated. The degree and pattern of glycosylation directly impact the ability to maintain a healthy intestine. The specific patterns of glycans expressed in the gut regulate a number of essential interactions between the host and its microbiota. For example, glycans serve as attachment sites for specific bacteria;^15^ glycan catabolism provides microbes with a carbon source, which impacts microbiota ecology;^16^ and glycan consumption by microbes influences the expression of microbial genes and metabolic pathways implicated in colonization and virulence.^17,18^ Alterations in glycosylation are associated with gut inflammation, active ulcerative colitis, Crohn’s disease, and colonic cancer, in human^19–22^ and murine models.^23–27^ The main determinants of gut glycan functional diversity are the various terminal epitopes that can be found on the glycan, in particular, fucose (mainly α1-2 linked fucose) and sialic acid (mainly α2-3 linked sialic acid).

Gut fucosylation is a protective mechanism for maintaining host-microbial symbiosis. α1-2 fucosylation of epithelial cells is induced by increased FUT2 (fucosyltransferase 2) expression, stimulated when Toll-like receptor ligands interact with the host immune system in the gut. FUT2 mediates the transfer of fucose to the terminal galactose on glycan chains of epithelial cell surface glycoproteins and glycolipids. Commensal bacteria cleave fucose from these fucosylated glycans, making free fucose available in the gut lumen. This fucose is then available to affect the expression of microbial metabolic pathways and reduce the expression of bacterial virulence genes.^26,28^ In particular, fucose sensing by pathogens and pathobionts represses expression of colitis-associated enterocyte effacement (LEE) virulence genes through activation of FusKR signal transduction.^29^ In the absence of proper gut fucosylation, beneficial symbionts are weakened and pathogenic bacteria strengthened, which leads to the breakdown of the epithelial barrier, microbial translocation, and gut inflammation.^30^ For example, homozygous mutation of FUT2 leads to altered microbiota and increased susceptibility to gut inflammatory diseases such as Crohn’s disease.^31,32^ Despite the importance of proper gut fucosylation as a protective mechanism against microbial translocation and inflammation, its role during HIV infection is not known.

Increased sialic acid catabolism (via sialidase) drives microbial dysbiosis and gut inflammation. Sialidases (neuraminidases) are the enzymes that catalyze the removal of terminal sialic acid residues from glycans. Glycans lacking sialic acid are termed hypo-sialylated. Increased sialidase activity drives microbial dysbiosis/translocation and gut inflammation by increasing the levels of free sialic acid, which is then available to support the growth of pathobionts, leading to microbial dysbiosis, microbial translocation, and gut inflammation.^33^ Sialidase originates from, among other sources, several species of the bacterial genus *Bacteroides*, including *B. fragilis*, *B. thetaiotaomicron*, and *B. vulgatus*.^33,34^ Some of these commensal bacterial strains are associated with inflammation during colitis and other inflammatory bowel diseases (IBD) in humans,^35–43^ and can induce inflammation and colitis in animal models.^44–47^ Furthermore, the ability of *B. vulgatus* to catabolize sialic acid during colitis induces intestinal inflammation by driving dysbiosis manifested by *Enterobacteriaceae* expansion.^33^ These reports support the notion that elevated sialic acid catabolism by the commensal gut microbiome may have a detrimental influence on the gut microenvironment.^43^ However, such an impact is yet to be examined during HIV infection.

Despite the fact that both an altered glycome and HIV infection have separately been associated with microbial translocation and gut inflammation, no study has investigated whether altered gut glycosylation contributes to HIV pathogenesis by intensifying HIV-associated microbial translocation and gut inflammation. We hypothesize that both fucosylation and sialylation play a role in HIV-associated microbial translocation and gut inflammation. This study is beginning to explore this hypothesis. We examined biopsy tissue from terminal ileum, right colon, and sigmoid colon, obtained from HIV-infected virally-suppressed individuals. We found that high levels of the detrimental hypo-sialylated T-antigen glycans and low levels of the beneficial α1-2 fucosylated glycans are associated with higher abundance of glycan-degrading microbial species (in particular, *Bacteroides vulgatus)*, a less diverse microbiome, higher levels of inflammation, and higher levels of ileum-associated HIV DNA. We also found that these associations are linked to the activation of the gut inflammasome-mediator eIF2 signaling pathway.

## Results

### The gut glycome is compartmentalized between the terminal ileum, right colon, and sigmoid colon of HIV+ ART-suppressed individuals

**Fig. 1:**
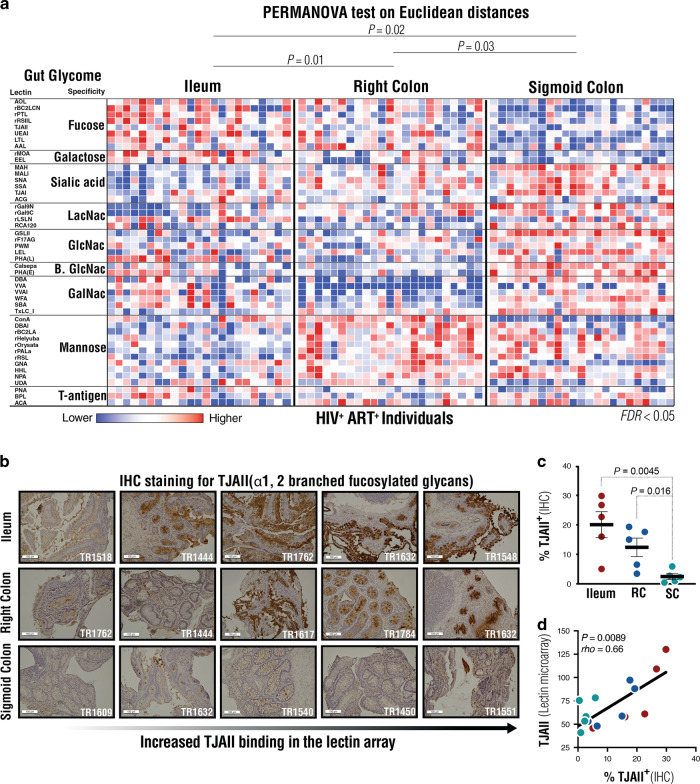
The gut glycome is compartmentalized between the terminal ileum, right colon, and sigmoid colon of HIV+ ART-suppressed individuals. **a** A heat-map representing relative binding of various glycans to lectins in three sites of the gut from 20 HIV+ ART-suppressed individuals. Heat colors show standardized Z-scores across samples; red indicates higher binding levels and blue indicates lower binding levels. ANOVA test and Permanova test on Euclidean distance were used for statistical analysis. **b** Representative immunohistochemistry (IHC) staining using TJAII lectin (binds to α1,2 fucosylated glycans) on ileum, right colon, and sigmoid colon samples (5 patients for each site), ordered from left to right by levels of lectin binding as assessed by the lectin array. The numbers in the lower right corner are patient IDs. **c** Analysis of IHC staining as percentage of tissue area positive for α1,2 fucosylation (binding to TJAII) in the three sites. Statistical comparisons were performed using a Mann–Whitney test. Lines and error bars represent mean and standard error of the mean (SEM). **d** Correlation between IHC and lectin array data, shown as relative binding for TJAII using the lectin microarray and percentage of area positive for TJAII staining using IHC, evaluated using Spearman’s rank correlation coefficient test.

We collected biopsies from the terminal ileum, right colon, and sigmoid colon of 20 HIV+ ART-suppressed individuals (clinical and demographic details are in Supplementary Table 1) and examined the glycosylation patterns using lectin microarray technology. The lectin microarray enables sensitive analysis of multiple glycan structures by employing a panel of immobilized lectins with known glycan-binding specificity, resulting in a specific “glycan signature” for each sample.^48,49^ In this study we used two versions of the lectin array, one with 96 lectins and one with 45 lectins (the specific lectins immobilized in each chip and their glycan-binding specificity are in Supplementary Tables 2 and 3, respectively). We found that the gut glycome was compartmentalized between the ileum and both right colon (*p* = 0.01, permanova test on Euclidean distance, Fig. 1a) and sigmoid colon (*p* = 0.02), as well as between the right and sigmoid colon (*p* = 0.03). Specifically, the binding of 47 lectins belonging to nine glycan categories differed between the three sites with false discovery rate (FDR) <0.05 (Fig. 1a). Within these nine categories, and relevant to our a priori hypotheses on fucosylated and sialylated glycans, levels of gut fucosylation decreased from ileum, to right colon, to sigmoid colon, whereas levels of gut sialylation increased in the same direction (Fig. 1a). To validate the quantitative abilities of the lectin microarray within the context of our study, we used immunohistochemistry (IHC) to stain sections of ileum, right colon, and sigmoid colon from a subset of the same individuals using the TJAII lectin (this lectin binds α1,2 fucosylated glycans). The staining intensity paralleled the quantitative values obtained by the lectin array (Fig. 1b–d). Similar IHC results were obtained using other lectins (UEAI, DBA, and HHL) (Supplementary Fig. 1).

### Gut microbial composition is associated with gut sialyation and fucosylation during ART-suppressed HIV infection

**Fig. 2:**
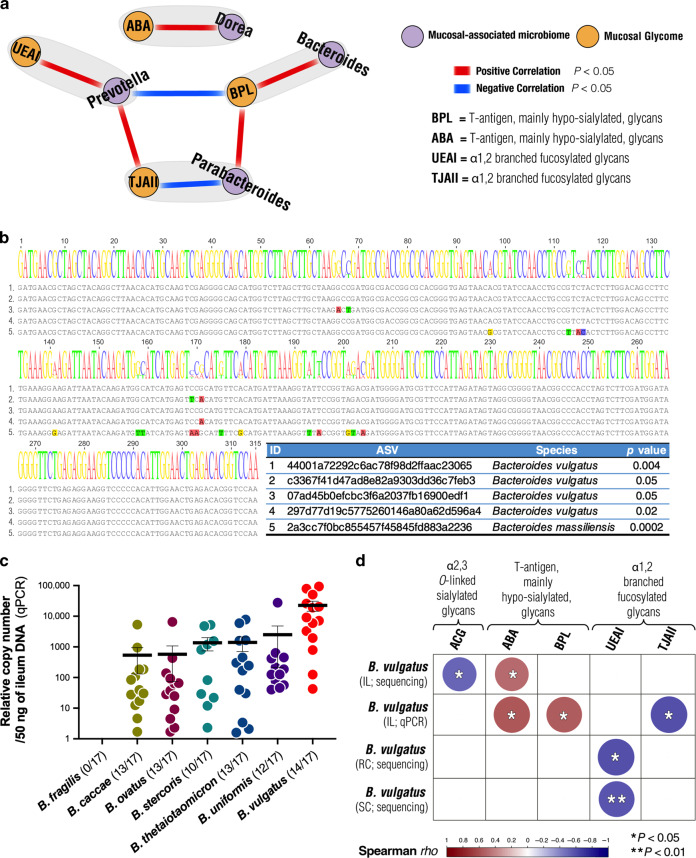
Levels of mucosal-associated *Bacteroides vulgatus* associate with gut sialyation and fucosylation during ART-suppressed HIV infection. **a** A network shows correlations between hypo-sialylated T-antigen or fucosylated glycans (orange circles) and members of the mucosal-associated microbiome (purple circles). Correlations were obtained by binning data from the three gut locations and using linear mixed effects model coefficient tests. Red lines represent positive correlations and blue lines represent negative correlations. **b** Sequence alignment of five ASVs consistent with *Bacteroides* species that were identified in the gut biopsies with >1% relative abundance across samples and found in >20% of the samples. **c** qPCR analysis of the relative copy number of seven different *Bacteroides* species in the ileum. Lines and error bars represent mean and SEM. The numbers in brackets show the total number of samples positive/total number of samples tested. **d** Heatmap shows the correlations between the levels of *Bacteroides vulgatus* and levels of sialylated, T-antigen (hypo-sialylated), and fucosylated glycans. Correlations were evaluated using Spearman’s rank correlation coefficient tests. IL, ileum; RC, right colon; SC, sigmoid colon. Nominal *p*-values are reported.

We examined the microbiome composition in the three gut sites from the 20 HIV+ ART-suppressed individuals using 16S rRNA marker gene sequencing. Using linear mixed effects model, we found four bacterial genera that correlated with levels of sialylated and/or fucosylated glycans (Fig. 2a; a comprehensive description of the associations between the gut glycome and the microbiome in each of the three sites is shown in Supplementary Fig. 2). Specifically, higher levels of *Bacteroides* (*p* = 0.009)*, Parabacteroides* (*p* = 0.009), and *Dorea* (*p* = 0.033), and lower levels of *Prevotella* (*p* = 0.039), correlated with higher levels of hypo-sialylated T-antigen glycans (Gal-GalNAc; measured by binding BPL and ABA lectins). Note that although ABA lectin is known to bind both sialylated and hypo-sialylated T-antigen, it is likely that ABA preferentially binds hypo-sialylated T-antigen glycans given that sialic acid catabolism (via sialidase) significantly enhances binding of ABA to colon cancer cell line (Supplementary Fig. 3). These correlations are compatible with the fact that several strains of *Bacteroides*, *Parabacteroides, and Dorea* release sialidase.^33,50–52^ Furthermore, increasing levels of *Parabacteroides* (*p* = 0.016) and decreasing levels of *Prevotella* (*p* < 0.038) correlated with lower levels of α1,2 branched fucosylated glycans (measured by binding to UEAI and TJAII lectins; Fig. 2a).

Focusing on each gut site separately (Supplementary Fig. 2A), we found that increasing levels of *Bacteroides* in the ileum correlated with higher levels of hypo-sialylated T-antigen glycans (binding to ABA and BPL lectins; *p* < 0.05). Consistently, increasing levels of the *Bacteroides* correlated with lower levels of α2,3 linked sialylated *O* glycans (measured by binding to ACG lectin; *p* < 0.05). The opposite relationships are observed with the *Prevotella* genus (*p* = 0.05), a *Bacteroides* antagonist.^53^ It is important to note that the correlation between levels of *Bacteroides* and sialylated *O* glycans was only observed in the ileum and not in the other two gut compartments (Supplementary Fig. 2).

*Bacteroides* is a taxonomically diverse genus with several species that are known to produce glycan-degrading enzymes and possibly contribute to the exacerbation of inflammation during IBD.^33–47^ In order to identify the *Bacteroides* species that are most abundant in our samples, and thus are potentiality responsible for the aforementioned correlations, we performed an additional analysis on the Amplicon Sequence Variants (ASVs) related to the *Bacteroides* species. We focused on species that have >1% relative abundance across samples and found in >20% of the samples. This analysis resulted in five ASVs (Fig. 2b). Using an in-house software, unassigner, (https://github.com/kylebittinger/unassigner), we found that 4/5 of these ASVs were consistent with *Bacteroides vulgatus*, and one with *Bacteroides massiliensis* (Fig. 2b). This relatively high abundance of *B. vulgatus* in our samples was further confirmed in the ileum using qPCR^54^ where we examined the relative abundance of seven different *Bacteroides* species (Fig. 2c). *B. vulgatus*^33,34,55–57^ and *B. massiliensis*,^58^ are capable of producing sialidase; *B. vulgatus* can also cleave fucose.^59,60^ Consistently, we found that higher levels of *B. vulgatus* correlated with lower levels of α2,3 linked sialylated *O* glycans (measured by binding to ACG; *p* = 0.024, *rho* = −0.507), higher levels of hypo-sialylated T-antigen (binding to ABA and BPL lectins; *p* < 0.039, *rho* > 0.5), and lower levels of α1,2 branched fucosylated glycans (measured by binding to TJAII lectin; *p* = 0.027, *rho* = −0.59), in the ileum (Fig. 2d). Additionally, we found that higher levels of *B. vulgatus* correlated with lower levels of α1,2 branched fucosylated glycans (measured by binding to UEAI lectin), in right colon (*p* = 0.027, *rho* = −0.59) and sigmoid colon (*p* = 0.009, *rho* = −0.56) (Fig. 2d).

These results suggest that microbial composition during ART-suppressed HIV infection, in particular the abundance of *B. vulgatus*, may impact gut sialylation and fucosylation during ART-suppressed HIV infection. Given that sialic acid catabolism and proper gut fucosylation play an important role in regulating microbial translocation and gut inflammation,^28–30,33^ we next examined the associations between gut sialylation or fucosylation and both microbial translocation and inflammation during ART-suppressed HIV infection.

### Gut hypo-sialylation is associated with lower microbial diversity, higher microbial translocation, and higher inflammation, during ART-suppressed HIV infection

We examined the associations between gut sialylated or hypo-sialylated glycans and microbial diversity and translocation, as well as, systemic inflammation.

#### Interactions between microbiome and inflammation

**Fig. 3:**
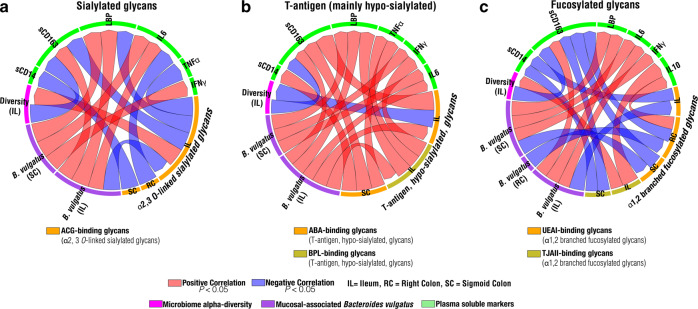
Gut glycosylation associates with markers of microbial dysbiosis, diversity, and translocation, as well as with markers of inflammation. Circos plots showing the correlation between **a** sialylated glycans, **b** hypo-sialylated T-antigen glycans, and **c** fucosylated glycans, and markers of microbial diversity and translocation, as well as, inflammation. Red lines represent positive correlations and blue lines represent negative correlations. Correlations were evaluated using Spearman’s rank correlation coefficient tests.

We used microbial alpha diversity as an index of healthy gut; microbial diversity is reduced during HIV infection.^12^ As expected, higher microbial alpha diversity (in the ileum) is associated with lower levels of plasma sCD163 (*p* = 0.015, *rho* = −0.54; Fig. 3a–c). sCD163 is a marker of LPS-induced inflammation; its levels independently predict mortality in HIV+ individuals.^61^ We also found that higher levels of *B. vulgatus* in the ileum correlated with higher microbial translocation and inflammation, as measured by plasma levels of LBP (lipopolysaccharide binding protein; *p* = 0.015*, rho* = 0.54*)*, sCD163 (*p* = 0.03*, rho* = 0.48), and IL6 (*p* = 0.04*, rho* = 0.46) (Fig. 3a–c). Furthermore, higher levels of *B. vulgatus* in the sigmoid colon correlated with higher LBP (*p* = 0.009*, rho* = 0.56), IFNγ (*p* = 0.025*, rho* = 0.5), and IL6 (*p* = 0.02*, rho* = 0.51) (Fig. 3a–c). In summary, in our sample population, lower microbial alpha diversity (in the ileum) and higher levels of *B. vulgatus* in gut biopsies are associated with higher levels of inflammation and microbial translocation during ART-suppressed HIV infection.

#### Interactions between sialylation and microbiome/inflammation

Focusing on sialylated glycans (binding to ACG lectin), we found that increased levels of mucosal-associated sialylated glycans correlated with higher ileum microbiome alpha diversity (*p* = 0.01, *rho* = 0.55) and lower markers of inflammation and microbial translocation (sCD163, sCD14, LBP, and TNFα; *p* ≤ 0.02*, rho* < *−*0.5, Fig. 3a). These correlations support our hypothesis that intact sialylated glycans convey a protective impact on the gut during ART-suppressed HIV infection. Focusing on hypo-sialylated T-antigen glycans (binding to ABA and/or BPL lectins), we found that increased levels of mucosal-associated hypo-sialylated T-antigen glycans correlated with lower ileum microbiome diversity (*p* = 0.001, *rho* = −0.68) and higher markers of inflammation and microbial translocation (sCD163, sCD14, LBP, TNFα, and IFNγ; *p* < 0.02*, rho* > 0.5, Fig. 3b). These correlations support our hypothesis that hypo-sialylated T-antigen glycans (a marker of increased sialic acid catabolism) are detrimental to the gut during ART-suppressed HIV infection.

### Gut fucosylation is associated with higher microbial diversity, lower microbial translocation, and lower inflammation, during ART-suppressed HIV infection

In addition to sialylation, we found that levels of mucosal-associated α1-2 fucose (binding to TJAII and UEAI lectins) correlated with higher ileum microbiome diversity (*p* = 0.03, *rho* = 0.48), lower plasma markers of inflammation and microbial translocation, i.e., sCD163 (*p* < 0.04, *rho* < −0.5) and sCD14 (*p* = 0.036, *rho* = −0.47), and higher plasma levels of the anti-inflammatory cytokine IL-10 (*p* < 0.04, *rho* > 0.5) (Fig. 3c). These correlations support our hypothesis that fucosylated glycans, convey a protective effect on the gut during ART-suppressed HIV infection.

### Levels of ileum hypo-sialylated T-antigen associate with levels of gut-associated HIV DNA

**Fig. 4:**
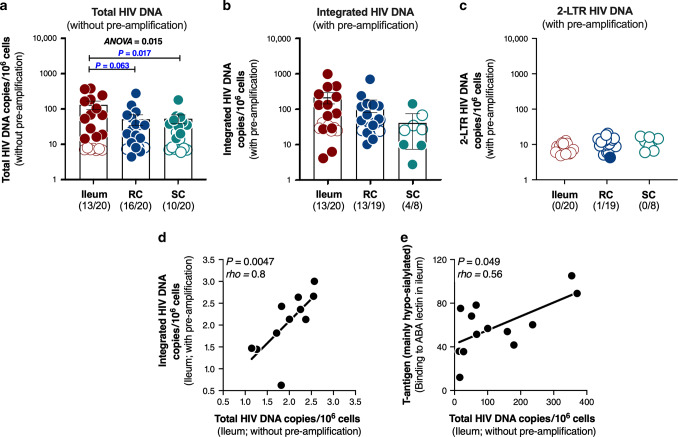
Levels of hypo-sialylated T-antigen glycans correlate with levels of total HIV DNA in the ileum of HIV+ ART-suppressed individuals. **a** Levels of total HIV DNA measured in the ileum, right colon, and sigmoid colon of HIV+ ART-suppressed individuals, using qPCR without a pre-amplification step. **b** Levels of integrated HIV DNA measured using qPCR after a pre-amplification step. **c** Levels of 2-LTR HIV DNA measured using qPCR after a pre-amplification step. The numbers in brackets show the total number of samples positive/total number of samples tested. Lines and error bars represent mean and SEM. Open circles represent limit of detection in samples with undetectable levels of HIV DNA. **d** Correlation between levels of total HIV DNA, measured in the ileum without a preamplification step, and levels of integrated HIV DNA, measured in the ileum after a preamplification step. Correlation was evaluated using Spearman’s rank correlation coefficient test. **e** Levels of hypo-sialylated T-antigen glycans correlate with levels of HIV DNA in the ileum. Correlation was evaluated using Spearman’s rank correlation coefficient test.

We next examined whether the detrimental impact of hypo-sialylated glycans, and/or the protective impact of fucosylated glycans, is extended to the levels of HIV DNA in this major organ for HIV persistence (the gut). First, we measured levels of total HIV DNA in the gut biopsies using qPCR with no pre-amplification step. We found that levels of total HIV DNA were higher in ileum than in the other two gut sites (Anova *p* = 0.015, Fig. 4a). Next, we measured levels of integrated HIV DNA (after pre-amplification with primers for genomic Alu elements and HIV sequences),^62^ and 2-LTR HIV DNA (unintegrated; after pre-amplification)^62^ in a subset of samples with excess DNA available. The majority of samples with detectable total HIV DNA also had detectable integrated HIV DNA, but the majority of samples were undetectable for 2-LTR HIV DNA (Fig. 4b, c). Levels of total HIV DNA, measured without pre-amplification, exhibited a strong positive correlation with levels of integrated HIV DNA, measured after pre-amplification (Fig. 4d, *p* = 0.0047, *rho* = 0.8). Together with the lack of detectable 2-LTR HIV DNA levels, these data suggest that the majority of HIV DNA measured in these gut biopsies is integrated. When we examined the association between the gut sialylation and levels of total HIV DNA, we found that increased levels of hypo-sialylated T-antigen glycans associated with higher levels of HIV DNA in the ileum (*p* = 0.049, *rho* = 0.56, Fig. 4e). No association was found for fucosylated glycans.

### Additional gut glycans correlate strongly with markers of inflammation and microbial translocation during ART-suppressed HIV infection

**Fig. 5:**
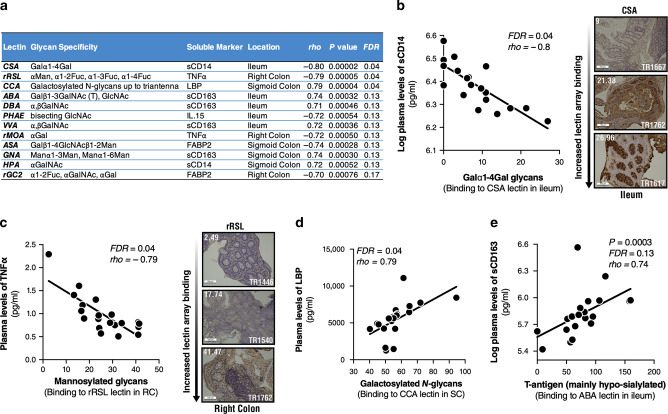
Levels of additional gut glycan structures also strongly correlate with markers of inflammation and microbial translocation during ART-suppressed HIV infection. **a** Table of glycan structures that associate with markers of inflammation and microbial translocation with an FDR < 0.2. Panels B-E show four exemplars. **b** Levels of Galα1-4Gal glycans (binding to CSA lectin) in the ileum correlated with lower levels of sCD14; representative IHC staining using the CSA lectin is also shown. Numbers in the lower right are patient IDs, and numbers in the top left corner are the relative binding values of CSA using the lectin microarray. **c** Levels of mannosylated glycans (binding to rRSL lectin) in the right colon correlated with lower levels of TNFα.; representative IHC staining using the rRSL lectin is also shown. **d** Levels of galactosylated glycans (binding to CCA lectin) in sigmoid colon correlated with higher levels of LBP. **e** Levels of hypo-sialylated T-antigen glycans (binding to ABA lectin) in the ileum associated with higher levels of sCD163. All correlations were evaluated using Spearman’s rank correlation coefficient tests.

In the previous results on sialylated and fucosylated glycans, we did not correct for multiple comparisons, given our a priori focus and hypotheses on fucosylated and sialylated glycans, as well as the exploratory nature of this pilot study. However, by taking advantage of the comprehensive analyses enabled by the lectin arrays, we were able to evaluate associations (with multiple comparisons accounted for) between other gut glycan structures and plasma markers of inflammation and microbial translocation during ART-suppressed HIV Infection. Twelve glycan structures correlated with markers of inflammation and microbial translocation with *FDR* < 0.2 (Fig. 5a). Of these, levels of Galα1-4Gal glycans in the ileum correlated with lower levels of sCD14 (*FDR* = 0.04, Fig. 5b), levels of mannosylated glycans in the right colon correlated with lower levels of TNFα (*FDR* = 0.04, Fig. 5c), levels of galactosylated glycans in sigmoid colon correlated with higher levels of LBP (*FDR* = 0.04, Fig. 5d), and as previously mentioned, levels of hypo-sialylated T-antigen glycans in the ileum correlated with higher levels of sCD163 (*FDR* = 0.13, Fig. 5d). These glycans represents additional targets that future studies could evaluate for their role in regulating microbial translocation and inflammation, during ART-suppressed HIV infection.

### The relationship between ileum-associated glycosylation and inflammation as well as HIV DNA is linked to the gut inflammasome-mediator eIF2 signaling pathway

Our data, so far, support our hypothesized detrimental effects of hypo-sialylated glycans and protective effects of fucosylated glycans on gut inflammation and HIV persistence during ART-suppressed infection. However, the host signals and pathways that were modulated by these processes are unclear. To begin elucidating host response signals and pathways, we performed a full transcriptomic analysis using RNAseq on the ileum samples (site of highest HIV DNA amounts) from the same 20 HIV+ ART-suppressed individuals. Samples from 15 individuals yielded a high-quality sequence data and were used for subsequent analyses.

**Fig. 6:**
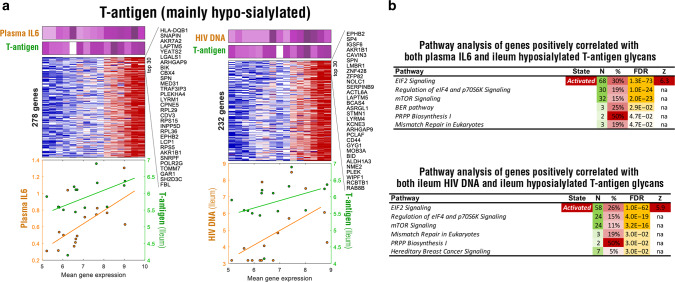
The association between hypo-sialylated T-antigen glycans and both inflammation and HIV DNA is linked to the activation of inflammasome-mediating eIF2 signaling. **a** Expression heat-maps and correlation plots of genes whose expression correlated positively with both levels of IL-6 in the plasma and levels of hypo-sialylated T-antigen glycans in the ileum (left), or genes whose expression correlated positively with both levels of HIV DNA and hypo-sialylated T-antigen glycans in the ileum (right). **b** Pathway analyses of the sets of genes in (**a**).

Focusing on hypo-sialylated T-antigen glycans, we identified 278 genes whose expression correlated positively with both levels of hypo-sialylated T-antigen glycans in the ileum and plasma levels of IL-6, a marker of inflammation (*p* < 0.05). Pathway analysis of these 278 genes revealed an activation of the translational initiator eukaryotic initiation factor 2 (eIF2) signaling pathway (*FDR* = 1.3E−73, *Z score* = 6.3). eIF2 signaling activation is known to induce the NLRP3 inflammasome in the gut.^63–65^ We also identified 232 genes whose expression correlated positively with both levels of hypo-sialylated T-antigen glycans and levels of HIV DNA in the ileum (*p* < 0.05). Pathway analysis of these 232 genes also revealed an activation of the eIF2 signaling pathway (*FDR* = 1.0E−62, *Z score* = 5.9) (Fig. 6). These results suggest that activation of inflammasome-mediating eIF2 signaling is involved in the detrimental impact of hypo-sialylated glycans on the gut during ART-suppressed HIV infection.

**Fig. 7:**
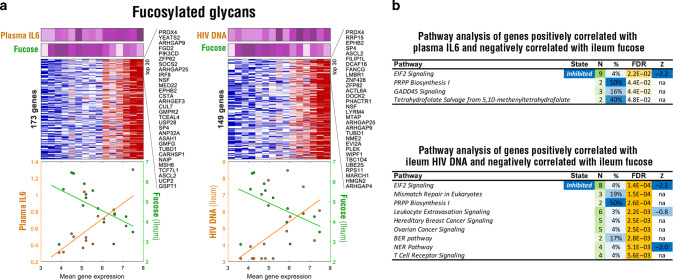
The association between fucosylated glycans and both inflammation and HIV DNA is linked to the inhibition of inflammasome-mediating eIF2 signaling. **a** Expression heat-maps and correlation plots of genes whose expression correlated positively with levels of IL-6 in the plasma and negatively with levels of fucosylated glycans in the ileum (left), or genes whose expression associated positively with levels of HIV DNA in the ileum and negatively with levels of fucosylated glycans in the ileum (right). **b** Pathway analyses of the sets of genes in (**a**).

Focusing on fucosylated glycans, we identified 173 genes whose expression correlated both negatively with levels of fucosylated glycans in the ileum and positively with plasma levels of IL-6 (*p* < 0.05). Pathway analysis of these 173 genes revealed inhibition of the eIF2 signaling pathway (*FDR* = 2.2E−02, *Z score* = −2.2). Finally, we identified 149 genes whose expression correlated both negatively with levels of fucosylated glycans in the ileum and positively with levels of ileum-associated HIV DNA (*p* < 0.05). Pathway analysis of these 149 genes also revealed inhibition of the eIF2 signaling pathway (*FDR* = 1.4E−04, *Z score* = −2.2) (Fig. 7). Consistent with these results, ileum samples with higher levels of hypo-sialylated T-antigen glycans and/or lower levels of fucosylated glycans (compared to median) show increases levels of the upstream regulators of eIF2 signaling (PERK and GCN2), and increases levels of the downstream inflammatory signaling pathways of eIF2 (NF-κB, LPS-induced TNFα, caspase-1, and MAPK/mTOR) (Supplementary Fig. 4). These combined results suggest a host signaling pathway that may explain both the detrimental effects of hypo-sialylated T-antigen glycans (activation of inflammasome-mediating eIF2 signaling), as well as the protective effects of fucosylated glycans (inhibition of inflammasome-mediating eIF2 signaling), in the gut during ART-suppressed HIV infection.

## Discussion

**Fig. 8:**
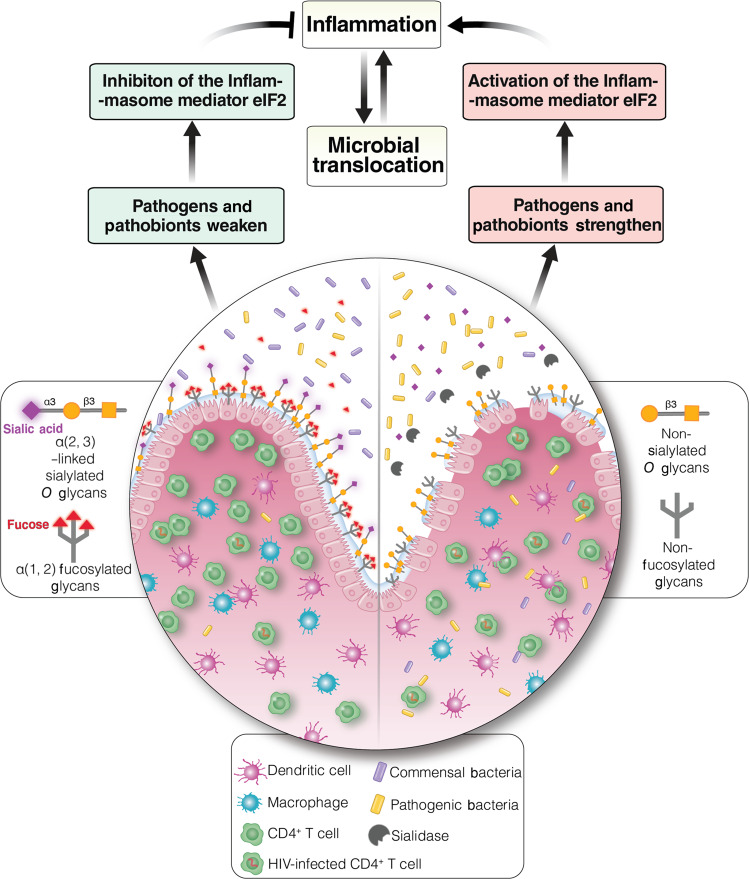
Proposed model of how glycomic patterns modulate microbial translocation and inflammation during ART-suppressed HIV infection. A proposed model on the role of gut sialylation and fucosylation in the host-microbe interplay during ART-suppressed HIV infection. Left. Healthy gut, with sialic acid attached to glycans and considerable fucosylation. Right. Pathological gut, with increased sialidase activity, increased free sialic acid, and lack of proper fucosylation. Pathogenic bacteria increase, epithelial barrier is compromised, microbes and their products translocate, and inflammation ensues.

We analyzed the glycome, microbiome, and transcriptome of gut biopsies (terminal ileum, right colon, and sigmoid colon) from HIV+ ART-suppressed individuals and linked these analyses to markers of microbial translocation and inflammation, as well as to levels of gut-associated HIV persistence. Our data support a model in which increased levels of sialic acid catabolism and lack of proper fucosylation may lead to higher microbial translocation and inflammation in the gut of HIV-infected individuals on suppressive ART. Our data further suggest that gut sialic acid catabolism and lack of proper fucosylation results in activation of the inflammasome-mediating eIF2 signaling, inducing inflammation, leading to microbial translocation, that in turn, feeds further inflammation (Fig. 8). Together our data suggest that this previously unappreciated factor, gut glycosylation, is a potential force that modulates the interactions between HIV infection, microbial translocation, and chronic inflammation. Given the importance of microbial translocation in shaping HIV disease progression, even during suppressive ART, understanding how specific glycan structures (especially fucosylated and hypo-sialylated T-antigen glycans) shape microbiota-gut interactions during HIV infection may help in designing strategies to mitigate these pathogenic mechanisms.

In the general population, gut glycomic alterations have been shown to not only correlate with microbial dysbiosis and translocation but also mechanistically drive it.^33,66,67^ In non-HIV mouse studies, oral administration of a sialidase inhibitor prevents microbial dysbiosis/translocation and decreases colitis severity.^33^ Similarly, oral treatment with L-fucose supplement in mice, reversed microbial dysbiosis caused by a high-fat diet, in both composition and function.^67^ In a recent study, L-fucose supplementation also ameliorated colitis in mice.^68^ In that study, and consistent with the results here, L-fucose inhibited the gut NLRP3 inflammasome in the gut.^68^ The NLRP3 inflammasome is known to be activated by the eIF2 signaling in epithelial cells and antigen-presenting cells (APCs), leading to production of inflammatory cytokines; inhibition of eIF2 signaling inhibits the NLRP3 inflammasome and prevents gut inflammation.^63–65^

Intriguing, our human data, using biopsies from HIV-infected ART-suppressed individuals, suggest that the link between higher levels of gut fucosylation and lower levels of microbial translocation and inflammation is associated with inhibition of the NLRP3 inflammasome-mediating eIF2 signaling, whereas the relation between hypo-sialylated T-antigen glycans and higher levels of microbial translocation, inflammation, and HIV DNA is associated with an activation of eIF2 signaling. Our data are the first to point to the potential role of gut inflammasome-mediating eIF2 signaling in regulating microbial translocation, inflammation, and persistence of HIV in the gut during ART-suppressed HIV infection. In addition, our findings suggest a potential mediator of this regulation, namely patterns of gut glycosylation. The vicious cycle in which HIV persistence causes inflammation that in turn contributes to HIV persistence has been suggested by many reports.^1–4,69,70^ Our results linking mucosal-associated HIV DNA levels to both gut hypo-sialylation and the activation of the gut inflammasome-mediator eIF2 signaling are intriguing, and further support the potential link between HIV persistence and the gut inflammatory milieu. Future studies examining the interplay between HIV infection/persistence, gut fucosylation, FUT2 expression, gut sialic acid catabolism, eIF2 signaling, and NLRP3 inflammasome, will provide even greater insights into the nature of glycan-mediated signaling in the gut during ART-suppressed HIV infection and will help define the opportunities for harnessing glycan-based therapeutics to reduce the burden of HIV-associated comorbidities and/or the levels of HIV persistence.

T-antigen levels are known to be increased in the mucin during colonic adenocarcinoma and ulcerative colitis.^19^ In our study, we found that the levels of hypo-sialylated T-antigen glycans associate with microbial translocation and inflammation during ART-suppressed HIV infection. We recently showed that HIV infection is associated with alterations, including hypo-sialylation, in the plasma circulating glycome; these changes were associated with markers of inflammation and levels of PBMCs-derived CD4+ T cell-associated HIV DNA and RNA.^71^ This HIV-associated hypo-sialylation is not completely reversed by ART.^71^ In the general population, hypo-sialylation on circulating glycoproteins drives inflammation and is associated with premature aging.^72–76^ The exact mechanism of this action is not clear. One suggestion is that an anti-inflammatory cascade is dampened: normally, sialic acid-containing glycans bind to sialic acid-binding proteins (siglecs) on the surface of monocytes/macrophages, which initiates an inhibitory signal that leads to an anti-inflammatory response, through inhibition of TLR4 signal transduction.^77–79^ Conceivably, HIV-mediated hypo-sialyation may interfere with this mechanism. In the gut, sialic acid catabolism has been shown to induce gut inflammation in mice by supporting the growth of pathobionts and pathogens.^33^ Our data also suggest a link between hypo-sialylation and the activation of inflammasome-mediating eIF2 signaling in the gut during HIV infection. Understanding the link between HIV-associated hypo-sialylation and inflammation, systematically, and within the gut, may provide clues about the mechanistic underpinnings of age- and inflammation-associated diseases in HIV-infected individuals, which persist despite viral suppression with ART.

In our study population, levels of mucosal-associated bacterial species, including *Bacteroides vulgatus*, are linked to higher levels of inflammation and microbial translocation during ART-suppressed HIV infection. Members of the *Bacteroides* genus in general and *B. vulgatus*, in particular, are prevalent commensal bacteria in the human gut;^80–83^ however some members of the *Bacteroides* (including *B. vulgatus*) are thought to play an important role in the exacerbation of inflammation during IBD.^35–37,47^ While there is controversy on the high abundance of *B. vulgatus* during Crohn’s disease and ulcerative colitis,^38–42,84^ it has been shown that some strains of *B. vulgatus* can induce colitis in mouse models.^44–47^ *B. vulgatus* can directly and indirectly induce several inflammatory pathways,^44–46,85,86^ albeit in a strain-dependent manner.^86–89^ *B. vulgatus* can also produce mucin-degrading enzymes, which could profoundly weaken the mucosal barrier function and strengthen pathogens.^33,34,45,55–57,90–92^ For example, the ability of *B. vulgatus* to catabolize sialic acid (via sialidase) during colitis induces intestinal inflammation by driving dysbiosis manifested by *Enterobacteriaceae* expansion.^33^ In addition to sialidase, *B. vulgatus* is known to cleave fucose causing a modification in the bacterial metabolic landscape that either decreases or increases inflammation caused by enteric infection.^29,59,60,93,94^ Future studies exploring the strain-level genome, transcriptome, and metabolome content of *B. vulgatus*, through metagenomics, metatranscriptomics, and metabolomics approaches, on both mucosal and feces-associated microbiome, will be needed to understand the precise impact of this bacterial species, and its carbohydrate metabolic pathways, on HIV-associated inflammation and microbial translocation.

For our study, we elected to examine the link between the gut glycome and the mucosal-associated microbiome as opposed to the feces-associated microbiome. These microbiomes differs significantly,^8,95^ and HIV-associated microbiome alterations seen in mucosal tissues are not always seen in feces from the same individual.^8^ We reasoned that bacteria associated with the mucosa are in close proximity to the gut glycome and therefore likely interact with it and the host immune system to a larger extent than do the bacteria associated with the feces. Nevertheless, examining the interplay between the gut glycome and both the feces-associated and mucosal-associated microbiomes may shed additional light on the interaction between the glycome and overall intestinal microbiome. We noted significant differences in the gut glycome among the different regions of the gut. In addition, most of the interactions between the gut glycome, microbiome, inflammation, and HIV DNA were observed more frequently in the terminal ileum than in the two sites of the colon. These results suggest that interactions between the gut glycome, microbiome, HIV, and the human immune system differ among the compartments of the gut. The small intestine has a thinner and more discontinuous mucus layer than the colon,^96,97^ and the ileum has abundant Peyer’s patches that contain large numbers of immune system cells. These features may be enabling more significant interactions between the microbiome and the host, during HIV infection, in this important gut compartment.

Our study has limitations. First, in our study, we elected to use the lectin microarray and IHC for glycomic analyses. Other, more traditional approaches, like mass spectrophotometry, can define exact glycan structures. However, these approaches usually require large samples, which is not feasible in many human studies. In our study, we examined the glycomes of biopsies from three sites of the gut of 20 HIV-infected ART-suppressed individuals, for the first time. Our lectin array data show similarities to studies that used mass spectrophotometry. For example, we showed a compartmentalization of the gut glycome across the three sites of the gut (Fig. 1). A mass spec study using samples from two HIV-negative individuals after autopsy showed the same directionality of compartmentalization.^96^ This, in addition to the validation with IHC, point to the accuracy of our lectin microarray analyses of the gut glycome. Second, gender, genetics, diet, and age, may impact gut glycosylation. Larger sample size and controlled animal studies will be needed to explore the full extent of the link between gut glycans and HIV, adjusting/controlling for all factors that can influence the glycome and microbiome. Third, given the exploratory nature of our study, multiple comparisons correction was not used for analyzing the microbiome data (Fig. 2). Validating our microbiome results using larger cohorts should be the subject of future studies. Lastly, our data describe cross-sectional samples during ART-suppressed HIV infection; there is a need to also analyze longitudinal changes (before and after HIV infection, and before and after ART) in animal models, with and without infection, and both pediatric and aged cohorts, in the future.

In summary, our findings represent the first proof-of-concept evidence that gut glycosylation may impact HIV-associated microbial translocation and inflammation, as well as HIV persistence, in the setting of ART suppression. Our data also implicate gut inflammasome-mediating eIF2 signaling, which has not been linked to HIV-associated complications in the gut. Although it is difficult to unequivocally demonstrate a causal relationship between the gut glycome, HIV-associated microbial translocation, and viral persistence, the robust literature from the inflammatory bowel disease field on the role of gut glycosylation in modulating microbiome composition and interaction with the host inflammasome is relevant to and consistent with our findings and hypotheses. This consistency suggests that the observed glycomic patterns and associated signals should be further explored for their potential significance as mediators of HIV-associated complications in the gut, using animal models of HIV infection. This current study as well as future studies on how HIV alters the gut glycome, despite suppressive ART, will advance our knowledge of the gut glycomic mechanistic underpinnings of HIV-associated inflammation and persistence. This knowledge can lay the groundwork for developing novel glycan-based therapeutic strategies to reduce the burden of HIV-associated microbial translocation/inflammation, e.g., using sialidase inhibitors, carbohydrate supplements, manipulating glycosyltransferases, or manipulating the link to eIF2 signaling. In addition, these studies are yielding and will continue to yield new information on the gut glycomic alterations associated with microbial translocation and inflammation. Thus, these findings are important not just for HIV, but also for other inflammation and premature aging diseases that affect the gastrointestinal tract.

## Methods

### Study cohorts

We examined the glycome, microbiome, and transcriptome of the ileum, right colon, and sigmoid colon biopsies from 20 HIV+ ART-suppressed individuals (Supplementary Table 1), that were collected at the Rush University Medical Center. Written informed consent was provided by all patients, and the protocol used was approved by the Institutional Review Board at the Rush University Medical Center (#12020204).

### Glycomic analysis using lectin microarrays

Two lectin microarrays were used in this study (Supplementary Tables 2 and 3). Gut biopsies were homogenized (Potter-Elvehjem homogenizer, Omni International) in the presence of Halt™ Protease Inhibitor Cocktail (ThermoFisher catalog #78415). Lysates were labeled with Cy3 dye and hybridized to the lectin microarrays. The resulting lectin chips were scanned for fluorescence intensity on each lectin-coated spot using an evanescent-field fluorescence scanner (GlycoTechnica Ltd.). All samples were run in triplicate and the average of the triplicate was used for analysis. Data were normalized using the global normalization method.

### Immunohistochemistry (IHC)

IHC was performed on formalin-fixed paraffin-embedded (FFPE) 4–5 μm serial sections. For fixation the tissue was placed in 10% buffered formalin for 6–12 h at 4 °C, followed by a PBS wash and stored in 70% EtOH at 4 °C until embedding. Fixed tissue samples were embedded in paraffin using a Leica Pyloris processor. Upon staining sections were deparaffinized and rehydrated followed by 1 min incubation in boiling antigen unmasking solution (H3301, Vector Laboratories). Blocking was performed with 2.5% Horse Serum for 1 h, followed by a streptavidin/biotin blocking step (SP-2002, Vector Laboratories). Furthermore, non-specific binding was blocked using Carbo-free™ blocking solution (SP-5040, Vector Laboratories). Between each step, the sections were washed in PBS. Biotin-conjugated lectins (National Institute of Advanced Industrial Science and Technology (AIST)) were individually optimized and added to the sections for 30 min followed by detection using Vectastain ABC peroxidase kit (PK-6100, Vector Laboratories). After lectin incubation and detection, the sections were washed in PBS + 0.05% Tween20. To visualize the sites of glycan expression, ImmPACT DAB (3,3’-diaminobenzidine) Substrate (SK-5105, Vector Laboratories) was added for an optimized time interval. All incubations were carried out at room temperature. Finally, sections were counterstained with hematoxylin and all slides were mounted with Permount (Electron Microscopy Sciences) and images were taken on a fluorescence microscope (Leica DM2000 LED, HC PL 20X FLUOTAR objective (NA 0.55)) using LAS 4.12 software. The frequencies of glycan (DAB) positive cell profiles were quantified using FIJI version 2.0.0-rc68/1.52i. After colour deconvolution (H DAB), the threshold for DAB was evaluated and set to 80 for all sections.

### Mucosal DNA and RNA extraction

Gut biopsies (from same individuals) were lysed in RLT Plus Buffer (Allprep isolation kit, Qiagen catalog #80204), and lysates were homogenized using the Pathogen Lysis Tubes S beads (Qiagen catalog #19091) on Qiagen TissueLyser II (6 min at 25 Hz). Total DNA and total RNA were extracted simultaneously from the lysates using the Allprep DNA/RNA/miRNA Universal Kit (Qiagen) with on-column DNase treatment (Qiagen RNase-Free DNase Set) during the RNA extraction. RNA quality was validated using the Agilent TapeStation, and the High Sensitivity RNA Screentape (Agilent, Santa Clara, CA) and quantity was determined using the Qubit 2·0 Fluorometer (ThermoFisher Scientific, Waltham, MA).

### Microbiome 16S rRNA marker gene sequencing

Mucosal-associated DNA from the three gut sites was processed using barcoded PCR primers annealing to the V1-V2 region of the 16S rRNA gene. PCR reactions were carried out in quadruplicate using Q5 High-Fidelity DNA Polymerase (NEB, Ipswich, MA). Each PCR reaction contained 0.5 μM of each primer, 0.34 U Q5 Pol, 1X Buffer, 0.2 mM dNTPs, and 5.0 μl DNA in a total volume of 25 μl. Cycling conditions were as follows: 1 cycle of 98 °C for 1 m; 30 cycles of 98 °C for 10 s, 56 °C for 20 s, and 72 °C for 20 s; 1 cycle of 72 °C for 8 min. Replicate reactions were combined, cleaned using SPRI beads (GE Healthcare Life Sciences), and quantified using PicoGreen (Thermo Fisher Scientific). An equimolar amount of each sample was pooled, and the resulting library was sequenced on the Illumina MiSeq using 2 × 250 bp chemistry. Extraction blanks and DNA free water were processed in parallel to allow for empirical assessment of environmental and reagent contamination. Positive controls, consisting of eight artificial 16S gene fragments, were also included (Integrated DNA Technologies).

### Microbiome 16S rRNA marker gene bioinformatics processing

16S rRNA marker gene sequences were processed using QIIME2 version 2017.11. After demultiplexing, the read pairs were truncated to 230 nucleotides length, denoised and merged using DADA2 software. The taxonomy of the resulting amplicon sequence variants (ASVs) of V1-V2 region of the bacterial 16s rRNA gene was identified using Naive-Bayes classifier trained on Green Genes database version 13_8. The unique sequences were aligned using MAFFT and a phylogenetic tree was built using FastTree. Bacterial species were identified from the ASVs using an in-house software called unassigner (https://github.com/kylebittinger/unassigner). Data files from QIIME2 were analyzed in the R environment.

### Relative quantification of *Bacteroides* species by qPCR

qPCR reactions using primers and probes sets specific for seven different species of *Bacteroides* were used to identify the most abundant *Bacteroides* species in the ileum biopsies. 50 ng of ileum DNA was used in a qPCR reaction contains 12.5 µl TaqMan Universal PCR master mix with UNG, 0.3 µM of each primer, and 0.2 µM TaqMan probe. Assays were performed in a QuantStudio 6 Flex Real-Time PCR System (Applied Biosystems) using the following protocol: one cycle of 95 °C for 10 min, followed by 40 cycles of a two-stage temperature profile of 95 °C for 15 s and 1 min at an optimal melting temperature of each primers/probe set, as previously described.^54^ Data were analyzed using the deltaCT method.

### RNA Sequencing

100 ng of DNAse treated total RNA was used to prepare library for Illumina sequencing using the Quant-Seq 3’mRNA-Seq Library Preparation Kit (Lexogen, Vienna, Austria). Library quantity was determined using qPCR (KAPA Biosystem, Wilmington, MA), and overall library size was resolved using the Agilent TapeStation and the DNA High Sensitivity D5000 ScreenTape (Agilent, Santa Clara, CA). Equimolar amount of libraries were pooled, denatured and High-Output, single read, 75 base pair Next Generation Sequencing was done on a NextSeq 500 (Illumina, San Diego, CA).

### RNASeq analysis

RNA-seq data were aligned using *bowtie2* against the hg19 version of the human genome, and *RSEM* v1.2.12 software was used to estimate raw read counts and RPKM using Ensemble transcriptome information and *DESeq2* was used for raw count normalization.^97–99^ Normalized expression of genes detected with at least 10 counts in one sample and non-zero counts in at least 5 samples were tested for correlations with levels glycans in the ileum, plasma levels of IL-6 and HIV DNA in the ileum using Spearman correlation and significantly associated genes passing *p* < 0.05 threshold were considered for enrichment analysis. Gene set enrichment analysis was done using QIAGEN’s Ingenuity® Pathway Analysis software (IPA®, QIAGEN Redwood City, www.qiagen.com/ingenuity; RRID: SCR_008653) using “Canonical pathways” category. Nominal *p*-values were adjusted for multiple testing using the Benjamini–Hochberg procedure to estimate false discovery rate (FDR).^100^ Select pathways that passed FDR < 5% and nominal *p* < 0.01 significance threshold and had at least five significant genes were reported. Predicted activation Z-score calculated by IPA based on direction of correlation between genes and glycans.

### qPCR quantification of mucosal-associated total HIV DNA

Total cellular HIV-1 DNA was quantified with a qPCR TaqMan assay using LTR-specific primers F522-43 (5’ GCC TCA ATA AAG CTT GCC TTG A 3’; HXB2522–543) and R626-43 (5’ GGG CGC CAC TGC TAG AGA 3’; 626–643) coupled with a FAM-BQ probe (5’ CCA GAG TCA CAC AAC AGA CGG GCA CA 3’) using the QuantStudio 6 Flex Real-Time PCR System (Applied Biosystems). Cell-associated HIV-1 DNA copy number was determined using a reaction volume of 20 μl with 10 μl of 2x TaqMan Universal Master Mix II, including UNG (Life Technologies), 4 pmol of each primer, 4 pmol of probe, and 5 μl of DNA. Cycling conditions were 50 °C for 2 min, 95 °C for 10 min, then 60 cycles of 95 °C for 15 s and 59 °C for 1 min. External quantitation standards were prepared from DNA isolated from ACH-2 cells in a background of HIV-1 negative human cellular DNA, calibrated to the Virology Quality Assurance (VQA, NIH Division of AIDS) cellular DNA quantitation standards. Cell counts were determined by qPCR using human genomic TERT (Life Technologies, Grand Island, NY). Specimens were assayed with up to 750 ng total cellular DNA in replicate reaction wells, and copy number was determined by extrapolation against a 7-point standard curve (1–10,000 copies) performed in triplicate.

### qPCR quantification of mucosal-associated integrated and 2-LTR HIV DNA

Integrated and 2-LTR HIV DNA copies were quantified using a two-step PCR reaction, as previously described.^62^ Integrated HIV DNA was pre-amplified with two Alu primers and a primer specific for the HIV LTR region, in addition to primers specific for the CD3 gene to determine cell count. 2-LTR HIV DNA was pre-amplified with primers specific to 2-LTR, in addition to primers specific for the CD3 gene to determine cell count. Nested qPCR was then used to amplify HIV and CD3 sequences from the first round of amplification. Specimens were assayed in triplicate. Integrated HIV DNA copy number was determined by extrapolation against a 5-point standard curve (3–30,000 copies), using extracted DNA from ACH-2 cells. 2-LTR HIV DNA copy number was determined by extrapolation against a 5-point standard curve (6–60,000 copies), using a plasmid contains the HIV 2-LTR region and part of the human CD3 gene.^62^

### Measurement of plasma markers of inflammation and microbial translocation

Plasma levels of IFNα, IFNγ, IL1RA, IL2, IL6, IL7, IL10, IL15, and TNFα were determined using MSD U‐PLEX multiplex assay (Meso Scale Diagnostic catalog # K15067L-1). Plasma levels of soluble CD14, soluble CD163, and lipopolysaccharide (LPS) binding protein (LBP), and FABP2 were quantified using Quantikine ELISA kits (R&D Systems, catalog #DC140, catalog #CD1630, catalog #DY870-05, and catalog # DFBP20, respectively).

### ABA staining of the SW480 colorectal cell line

The SW480 cells were provided as a gift from Drs. Meenhard Herlyn and Rajasekharan Somasundaram at the Wistar institute.Sialidase was prepared in house using *Vibrio cholerae nanH* gene cloned into pCVD364 vector, that was generously provided to us by Dr. Eric R. Vimr at University of Illinois Urbana.^101^ Cells were either treated with 50 µg/ml of sialidase or PBS as control and incubated at 37 °C for 1 h. Cells were then washed twice, stained with FITC-conjugated ABA lectin (Vector Laboratories, Inc.), and analyzed for PE fluorescence using LSR II flow cytometer and FACSDiva software (Becton Dickinson). Data were analyzed using FlowJo (TreeStar Inc.).

### Statistical analysis

For each sample, alpha diversity of the microbial community was calculated using Shannon diversity metric. Relative abundance of a taxon was defined as the number of amplicon sequence variants (ASVs) assigned to the taxon divided by the total number in the sample. Correlation between the alpha diversity and relative abundance of bacterial taxa with the lectin levels and plasma cytokine markers were calculated using linear mixed effects model for tissues combined or Spearman correlation for each tissue type separately. To calculate the difference in lectin levels across the GI tract, liner mixed effects models were used where the GI location was used as fixed effect and the subject IDs were used as random effect. To compare the cumulative lectin profiles between tissue types, PERMANOVA test on Euclidean distances between samples was used. The p-values were corrected for false discovery rate (FDR) using the method of Benjamini and Hochberg where indicated.

## Supplementary information

Supplementary Figures and Tables
