# Supplementary material for: Sialylation and fucosylation modulate inflammasome-activating eIF2 Signaling and microbial translocation during HIV infection
Source: Mucosal Immunol. 2020 Mar 9;13(5):753–66. doi: 10.1038/s41385-020-0279-5 (PMC7434596; doi:10.1038/s41385-020-0279-5)
Supplement: Supplementary file 1 — Supplementary Figures and Tables [file 41385_2020_279_MOESM1_ESM.pdf]

Supplementary Figure 1

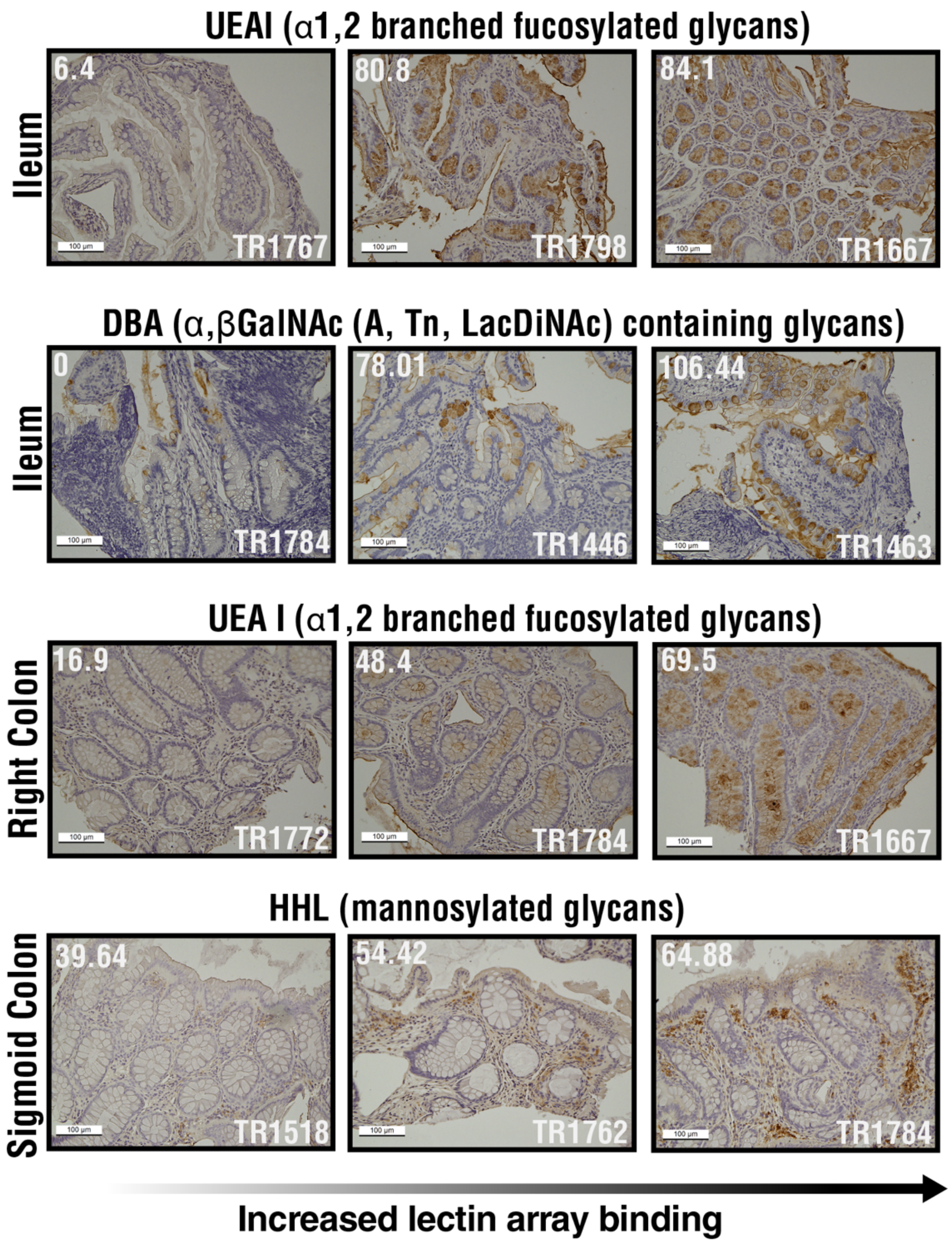

Supplementary Figure 1. Representative staining of UEAI, DBA, and HHL lectins using IHC. Numbers in the lower right corner are the patient IDs, and numbers in the top left corner are the relative binding values of these lectins using the lectin microarray.

Supplementary Figure 2

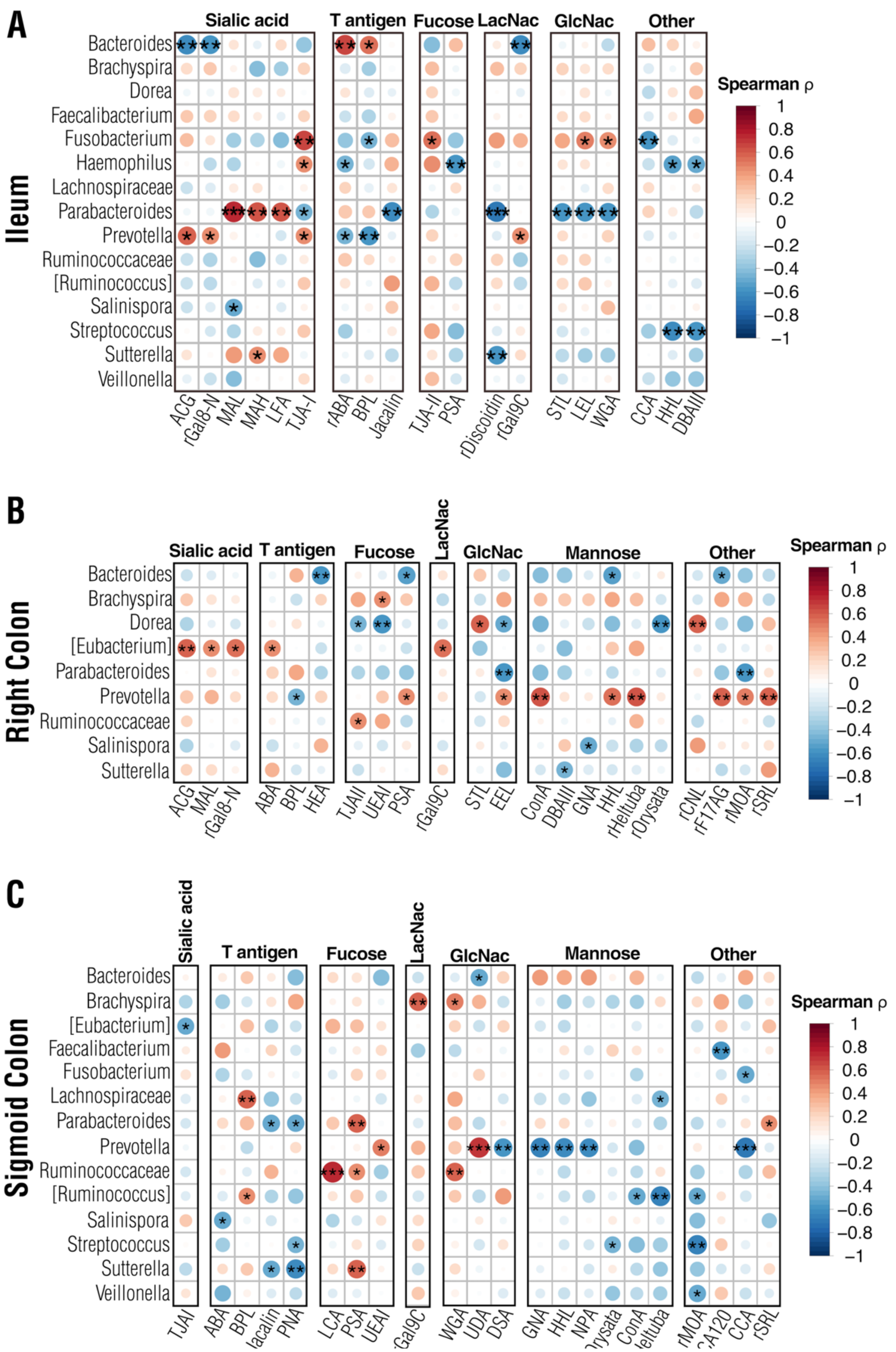

Supplementary Figure 2. Heat-maps representing correlations between the gut glycome and the gut microbiome separated per site: **(A)** Ileum, **(B)** Right colon, and **(C)** sigmoid colon. Heat colors show spearman r coefficients; red indicates a positive correlation, and blue indicates a negative correlation. * = p<0.05; ** = p<0.01; and *** = p<0.001. All correlations were evaluated using Spearman's rank correlation coefficient tests.

Supplementary Figure 3

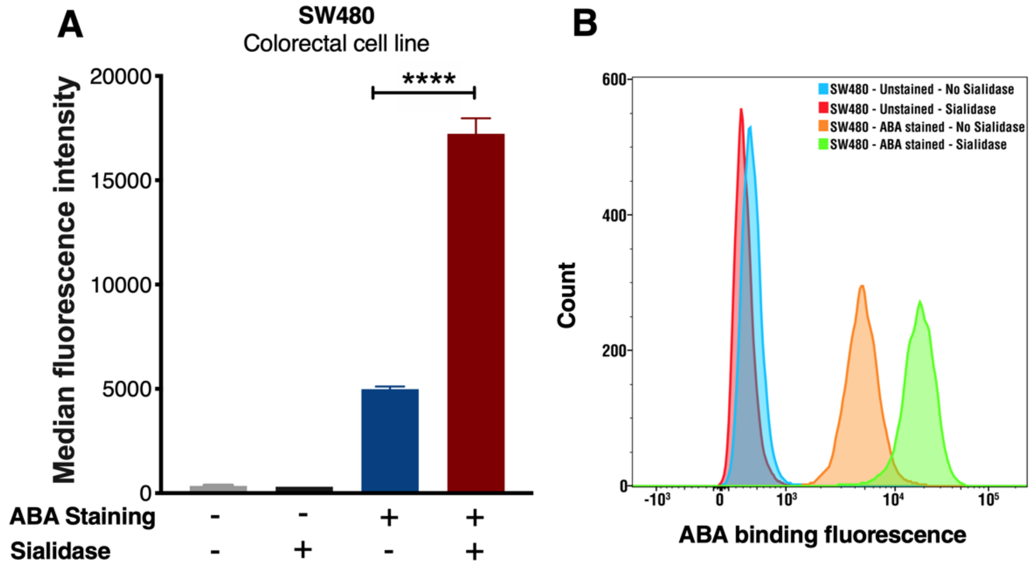

Supplementary Figure 3. ABA preferably binds to hypo-sialylated T-antigen glycans. SW480 colorectal cell line were stained with ABA in the presence of absence of 50μg/ml sialidase. **(A)** Median fluorescence intensity of ABA binding is higher when cells treated with sialidase compared to controls. Unpaired t-test. Lines and error bars represent mean and SEM. **** = $p<0.0001$. **(B)** A representative example of data in panel A.

Supplementary Figure 4

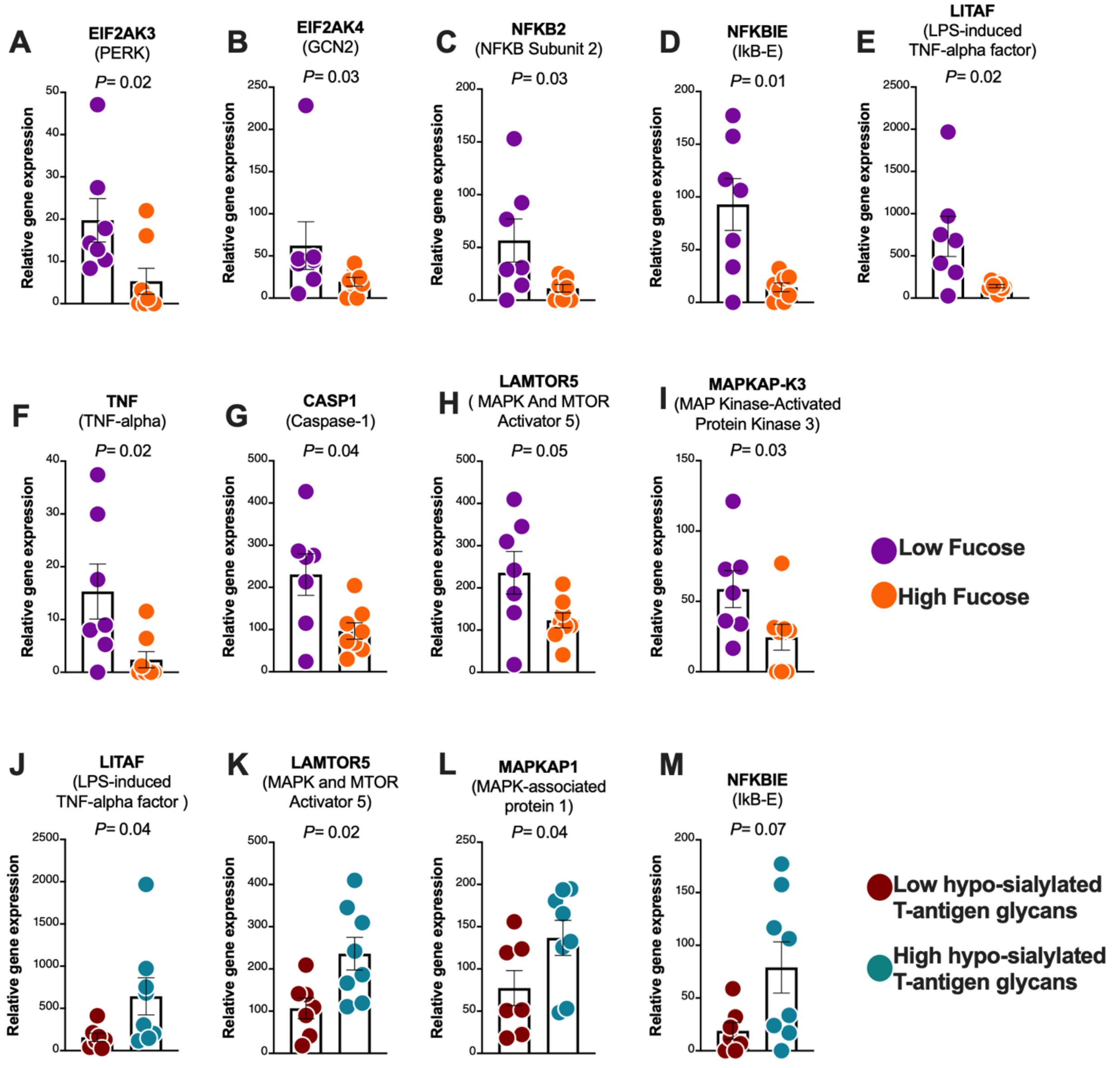

Supplementary Figure 4. High levels of hypo-sialylated T-antigen glycans and low levels of fucosylated glycans associate with higher levels of upstream regulators and downstream signaling pathways of eIF2. Ileum samples were divided to high and low levels of hypo-sialylated T-antigen glycans (compared to median binding to ABA lectin) or high and low levels of α1-2 fucosylated glycans (compared to median binding to UEAI lectin). Gene expression levels of the indicated genes were compared between the groups using the Mann–Whitney t-test. Lines and error bars represent mean and SEM.

Supplementary Table 1. Demographic and clinical data of the study participants.

Donor ID	CD4 count (cells/mm3)	VL (copies/ml)	Gender†	Race¥	Age (years)	BMI	Active hepatitis co-infection	Current antiretroviral therapy	Other mediciation(s)	Current Antibiotics	Cancer	Gastrointestinal Disorder	Dysmetabolic syndrome	Current Alcohol User
TR1551	178	<40	F	AA	53	25.04	No	Yes, Not documented	No	Not documented	No	Grade I internal hemorrhoids	No	Not documented
TR1762	272	<40	F	AA	54	27.87	No	Yes, Not documented	No	Not documented	No	Not documented	No	Not documented
TR1798	1159	<40	F	AA	54	28.36	No	Efavirenz/Emtricitabine /Eenofovir	Amlodipine	No	No	Treated Hepatitis B	Hypertension	Not documented
TR1767	390	<40	F	AA	57	29.62	No	Yes, Not documented	No	Not documented	No	Hemorrhoids/constipation	No	Yes
TR1667	739	<40	M	AA	49	24.42	No	Emtricitabine/Eenofovir, Atazanavir/Ritonavir	Cymbalta, Robitussin Ac, Benzonatate	Yes - azithromycin	No	Sigmoid diverticulum	Congestive Heart Failure	Yes
TR1632	669	<40	M	AA	50	22.02	No	Yes, Not documented	No	Not documented	No	Not documented	No	Not documented
TR1617	521	<40	M	AA	50	25.66	No	Efavirenz, Emtricitabine, and Tenofovir Disoproxil Fumarate	No	No	No	Small intestine surgery	No	Not documented
TR1548	452	<40	M	AA	50	27.88	No	Yes, Not documented	No	Not documented	No	Not documented	No	Not documented
TR1629	346	<40	M	AA	51	22.58	No	Yes, Not documented	No	No	No	Not documented	No	Yes
TR1609	508	<40	M	AA	51	23.35	No	Efavirenz, Emtricitabine, and Tenofovir Disoproxil Fumarate	Pravachol	No	No	Internal hemorrhoids	No	Not documented
TR1540	319	<40	M	AA	51	25.8	No	Efavirenz, Emtricitabine, and Tenofovir Disoproxil Fumarate	No	No	No	Internal hemorrhoids	No	Yes
TR1463	381	<40	M	AA	54	28.39	No	Yes, Not documented	Pravastatin	Not documented	No	Internal hemorrhoids, diverticulosis, treated Hepatitis C	Heart Disease, Hypertension	Not documented
TR1784	407	<40	M	AA	54	28.7	No	Yes, Not documented	Protonix, Levothyroxine, Centrum silver pro, Metamucil	No	No	Not documented	No	Not documented
TR1450	405	<40	M	AA	58	21.9	No	Yes, Not documented	No	No	No	Internal hemorrhoids	Hypertension	Yes
TR1772	526	<40	M	C	51	29.71	No	Efavirenz, Emtricitabine, and Tenofovir Disoproxil Fumarate	Asprin	No	No	viral gastroenteritis	Kidney Disease	Not documented
TR1518	789	<40	M	HA	35	15.16	No	Yes, Not documented	No	No	No	Anal fistula, Submucosal rectal abscess	No	No
TR1444	118	<40	M	HA	48	33.46	No	Efavirenz, Emtricitabine, and Tenofovir Disoproxil Fumarate	No	Not documented	No	Abdominal pain	No	Not documented
TR1446	303	<40	M	HA	51	26.94	No	Yes, Not documented	No	No	No	Abdominal surgery	No	No (former user)
TR1562	516	<40	F	C/HA	54	25.59	No	Emtricitabine/Tenofovir, Abacavir	No	No	No	Mild diverticulosis in sigmoid colon	No	No
TR1778	999	<40	M	AA	59	21.29	No	Yes, Not documented	Losartan	No	No	Irritable bowel syndrome, treated Hepatitis B	Hypertension,Type-2 Diabetes, Chronic Obstructive Pulmonary Disease, Coronary Artery Disease, High Cholesterol, Chronic Kidney Disease	No (former user)

Note that none of the 20 donors had asthma, bronchitis, pneumonia, bleeding disorders, skin disorders, recent infectious disease, mobility limitations, or mental illness, at the time of the sample collection.

† M = male, F = female

¥AA = African American, C = Caucasian, HA = Hispanic American

Supplementary Table 2. Lectins used for 96 lectin microarray [1]

	Name	Species	Origin	Glycan specificity[2]
1	LFA	*Limax flavus*	Natural	Sia
2	WGA	*Triticum vulgaris*	Natural	(GlcNAc)n, polySia
3	PVL	*Psathyrella velutina*	Natural	Sia, GlcNAc
4	MAL	*Maackia amurensis*	Natural	α2-3Sia
5	MAH	*Maackia amurensis*	Natural	α2-3Sia
6	ACG	*Agrocybe cylindracea*	Natural	α2-3Sia
7	rACG	*Agrocybe cylindracea*	*Recombinant*	α2-3Sia
8	rGal8N	*Homo sapiens*	*Recombinant*	α2-3Sia
9	SNA	*Sambucus nigra*	Natural	α2-6Sia
10	SSA	*Sambucus sieboldiana*	Natural	α2-6Sia
11	TJAI	*Trichosanthes japonica*	Natural	α2-6Sia
12	rPSL1a	*Polyporus squamosus*	*Recombinant*	α2-6Sia
13	PHAL	*Phaseolus vulgaris*	Natural	GlcNAcβ1-6Man (Tetraantenna)
14	DSA	*Datura stramonium*	Natural	GlcNAcβ1-6Man (Tetraantenna)
15	TxLcI	*Tulipa gesneriana*	Natural	Galactosylated N-glycans up to triantenna
16	ECA	*Erythrina cristagalli*	Natural	βGal
17	RCA120	*Ricinus communis*	Natural	βGal
18	rGal7	*Homo sapiens*	*Recombinant*	Type1 LacNAc, chondroitin polymer
19	rGal9N	*Homo sapiens*	*Recombinant*	GalNAcα1-4Gal (A), PolyLacNAc
20	rGal9C	*Homo sapiens*	*Recombinant*	PolyLacNAc, Branched LacNAc
21	rC14	*Gallus gallus domesticus*	*Recombinant*	Branched LacNAc
22	rDiscoidin II	*Dictyostelium dicodeum*	*Recombinant*	LacNAc, Galβ1-3GalNAc (T), GalNAc (Tn)
23	BPL	*Bauhinia purpurea alba*	Natural	Galβ1-3GlcNAc(GalNAc), α/βGalNAc
24	rCGL2	*Homo sapiens*	*Recombinant*	GalNAcα1-3Gal (A), PolyLacNAc
25	PHAE	*Phaseolus vulgaris*	Natural	bisecting GlcNAc
26	GSLII	*Griffonia simplicifolia*	Natural	GlcNAcβ1-4Man
27	rSRL	*Sclerotium rolfsii*	*Recombinant*	Core1,3, agalacto N-glycan
28	UDA	*Urtica dioica*	Natural	(GlcNAc)n
29	PWM	*Phytolacca americana*	Natural	(GlcNAc)n
30	rF17AG	*Escherichia coli*	*Recombinant*	GlcNAc
31	rGRFT	*Griffithia sp.*	*Recombinant*	Man
32	NPA	*Narcissus pseudonarcissus*	Natural	Manα1-3Man
33	ConA	*Canavalia ensiformis*	Natural	M3, Manα1-2Manα1-3(Manα1-6)Man, GlcNAcβ1-2Manα1-3(Manα1-6)Man
34	GNA	*Galanthus nivalis*	Natural	Manα1-3Man, Manα1-6Man
35	HHL	*Hippeastrum hybrid*	Natural	Manα1-3Man, Manα1-6Man
36	ASA	*Allium sativum*	Natural	Galβ1-4GlcNAcβ1-2Man
37	DBAI	*Dioscorea batatas*	Natural	High-man
38	CCA	*Castanea crenata*	Natural	Galactosylated N-glycans up to triantenna
39	Heltuba	*Helianthus t uberosus*	Natural	Manα1-3Man
40	rHeltuba	*Helianthus tuberosus*	*Recombinant*	Manα1-3Man
41	ADA	*Allomyrina dichtoma*	Natural	α2-6Sia, Forssman, A, B
42	VVAII	*Vicia villosa*	Natural	Man, Agalacto
43	rOrysata	*Oryza sativa*	*Recombinant*	Manα1-3Man, Highman, biantenna
44	rPALa	*Phlebodium aureum*	*Recombinant*	Man5, biantenna
45	rBanana	*Musa acuminata*	*Recombinant*	Manα1-2Manα1-3(6)Man
46	rCalsepa	*Calystegia sepium*	*Recombinant*	Biantenna with bisecting GlcNAc
47	rRSL	*Ralstonia solanacearum*	*Recombinant*	αMan, α1-2Fuc (H), α1-3Fuc (Le^x), α1-4Fuc (Le^a)
48	rBC2LA	*Burkholderia cenocepacia*	*Recombinant*	αMan, High-man
49	AOL	*Aspergillus oryzae*	Natural	α1-6Fuc (Core), α1-2Fuc (H), α1-3Fuc (Le^x), α1-3Fuc (Le^a)
50	AAL	*Aleuria aurantia*	Natural	α1-6Fuc (Core), α1-2Fuc (H), α1-3Fuc (Le^x), α1-3Fuc (Le^a)
51	rAAL	*Aleuria aurantia*	*Recombinant*	α1-6Fuc (Core), α1-2Fuc (H), α1-3Fuc (Le^x), α1-3Fuc (Le^a)
52	rPAIIL	*Pseudomonas aeruginosa*	*Recombinant*	αMan, α1-2Fuc (H), α1-3Fuc (Le^x), α1-4Fuc (Le^a)
53	rRSIIL	*Ralstonia solanacearum*	*Recombinant*	α1-2Fuc (H), α1-3Fuc (Le^x), α1-3Fuc (Le^a)
54	rPTL	*Pholiota terrestris*	*Recombinant*	α1-6Fuc
55	PSA	*Pisum sativum*	Natural	α1-6Fuc up to biantenna
56	LCA	*Lens culinaris*	Natural	α1-6Fuc up to biantenna
57	rAOL	*Aspergillus oryzae*	*Recombinant*	α1-2Fuc (H), α1-3Fuc (Lex), α1-3Fuc (Lea)
58	rBC2LCN	*Burkholderia cenocepacia*	*Recombinant*	Fuc α1-2Galb1-3GlcNAc (GalNAc)
59	LTL	*Lotus tetragonolobus*	Natural	Fuc (Le^x, Le^y)
60	UEAI	*Ulex europaeus*	Natural	α1-2Fuc
61	TJAII	*Trichosanthes japonica*	Natural	α1-2Fuc
62	MCA	*Momordica charantia*	Natural	α1-2Fuc
63	GSLI	*Griffonia simplicifolia*	Natural	αGalNAc (A, Tn), αGal (B)
64	PTLI	*Psophocarpus tetragonolobus*	Natural	αGalNAc (A, Tn)
65	GSLIA4	*Griffonia simplicifolia*	Natural	αGalNAc (A, Tn)
66	rGC2	*Geodia cydonium*	*Recombinant*	α1-2Fuc (H), αGalNAc (A), αGal (B)
67	GSLIB4	*Griffonia simplicifolia*	Natural	αGal (B)
68	rMOA	*Marasmius oreades*	*Recombinant*	αGal (B)
69	EEL	*Euonymus europaeus*	Natural	αGal (B)
70	rPAIL	*Pseudomonas aeruginosa*	*Recombinant*	α,βGal, αGalNAc (Tn)
71	LEL	*Lycopersicon esculentum*	Natural	Polylactosamine, (GlcNAc)n
72	STL	*Solanum tuberosum*	Natural	Polylactosamine, (GlcNAc)n
73	rGal3C	*Homo sapiens*	*Recombinant*	LacNAc, polylactosamine
74	rLSLN	*Laetiporus sulphureus*	*Recombinant*	LacNAc, polylactosamine
75	rCGL3	*Coprinopsis cinerea*	*Recombinant*	LacDiNAc
76	PNA	*Arachis hypogaea*	Natural	Galβ1-3GalNAc (T)
77	ACA	*Amaranthus caudatus*	Natural	Galβ1-3GalNAc (T)
78	HEA	*Hericium erinaceum*	Natural	Galβ1-3GalNAc (T)
79	ABA	*Agarics bisporus*	Natural	Galβ1-3GalNAc (T), GlcNAc
80	Jacalin	*Artocarpus integrifolia*	Natural	Galβ1-3GalNAc (T), GalNAcα (Tn)
81	MPA	*Maclura pomifera*	Natural	Galβ1-3GalNAc (T), GalNAcα (Tn)
82	HPA	*Helix pomatia*	Natural	αGalNAc (A, Tn)
83	VVA	*Vicia villosa*	Natural	α,βGalNAc (A, Tn, LacDiNAc)
84	DBA	*Dolichos biflorus*	Natural	α,βGalNAc (A, Tn, LacDiNAc)
85	SBA	*Glycine max*	Natural	α,βGalNAc (A, Tn, LacDiNAc)
86	rPPL	*Pleurocybella porrigens*	*Recombinant*	α,βGalNAc (A, Tn, LacDiNAc)
87	rCNL	*Clitocybe nebularis*	*Recombinant*	α,βGalNAc (A, Tn, LacDiNAc)
88	rXCL	*Xerocomus chrysenteron*	*Recombinant*	Core1,3, agalacto N-glycan
89	VVA I	*Vicia villosa*	Natural	GalNAcβ1-3(4)Gal
90	WFA	*Wisteria floribunda*	Natural	Terminal GalNAc, LacDiNAc
91	rABA	*Agarics bisporus*	*Recombinant*	Galβ1-3GalNAc (T), GlcNAc
92	rDiscoidin I	*Dictyostelium Discodeum*	*Recombinant*	Gal
93	DBAIII	*Dioscorea batatas*	Natural	Maltose
94	rMalectin	*Homo sapiens*	*Recombinant*	Glcα1-2Glc
95	CSA	*Oncorhynchus keta*	Natural	Rhamnose, Galα1-4Gal
96	FLAG-EW29Ch-E20	*Lumbricus terrestris*	*Recombinant*	6-sulfo-Gal

[1]Abbreviations: Gal (D-galactose), GalNAc (N-acetyl-galactosamine), GlcNAc (N-acetyl-glucosamine), Fuc (L-fucose), Glc (D-glucose), Sia (Sialic acid), LacNAc (N-acetyl-lactosamine).

[2]Specificity data was obtained by frontal affinity chromatography and glycoconjugate microarray.

Supplementary Table 3. Lectins used for 45 lectin microarray [1]

	Name	Species	Origin	Glycan specificity[2]
1	LTL	*Lotus tetragonolobus*	*Natural*	Fuc (Le^x, Le^y)
2	PSA	*Pisum sativum*	*Natural*	α1-6Fuc up to biantenna
3	LCA	*Lens culinaris*	*Natural*	α1-6Fuc up to biantenna
4	UEAI	*Ulex europaeus*	Natural	α1-2Fuc
5	AOL	*Aspergillus oryzae*	Recombinant	α1-6Fuc (Core), α1-2Fuc (H), α1-3Fuc (Le^x), α1-3Fuc (Le^a)
6	AAL	*Aleuria aurantia*	Natural	α1-6Fuc (Core), α1-2Fuc (H), α1-3Fuc (Le^x), α1-3Fuc (Le^a)
7	MAL	*Maackia amurensis*	*Natural*	α2-3Sia
8	SNA	*Sambucus nigra*	Natural	α2-6Sia
9	SSA	*Sambucus sieboldiana*	Natural	α2-6Sia
10	TJAI	*Trichosanthes japonica*	Natural	α2-6Sia
11	PHAL	*Phaseolus vulgaris*	Natural	GlcNAcβ1-6Man (Tetraantenna)
12	ECA	*Erythrina cristagalli*	Natural	βGal
13	RCA120	*Ricinus communis*	Natural	βGal
14	PHAE	*Phaseolus vulgaris*	*Natural*	bisecting GlcNAc
15	DSA	*Datura stramonium*	Natural	GlcNAcβ1-6Man (Tetraantenna)
16	GSLII	*Griffonia simplicifolia*	Natural	GlcNAcβ1-4Man
17	NPA	*Narcissus pseudonarcissus*	Natural	Manα1-3Man
18	ConA	*Canavalia ensiformis*	*Natural*	M3, Manα1-2Manα1-3(Manα1-6)Man, GlcNAcβ1-2Manα1-3(Manα1-6)Man
19	GNA	*Galanthus nivalis*	Natural	Manα1-3Man, Manα1-6Man
20	HHL	*Hippeastrum hybrid*	Natural	Manα1-3Man, Manα1-6Man
21	ACG	*Agrocybe cylindracea*	Natural	α2-3Sia
22	TxLcI	*Tulipa gesneriana*	*Natural*	Galactosylated N-glycans up to triantenna
23	BPL	*Bauhinia purpurea alba*	Natural	Galβ1-3GlcNAc(GalNAc), α/βGalNAc
24	TJAII	*Trichosanthes japonica*	Natural	α1-2Fuc
25	EEL	*Euonymus europaeus*	*Natural*	αGal (B)
26	ABA	*Agarics bisporus*	Natural	Galβ1-3GalNAc (T), GlcNAc
27	LEL	*Lycopersicon esculentum*	*Natural*	Polylactosamine, (GlcNAc)n
28	STL	*Solanum tuberosum*	Natural	Polylactosamine, (GlcNAc)n
29	UDA	*Urtica dioica*	Natural	(GlcNAc)n
30	PWM	*Phytolacca americana*	Natural	(GlcNAc)n
31	Jacalin	*Artocarpus integrifolia*	*Natural*	Galβ1-3GalNAc (T), GalNAcα (Tn)
32	PNA	*Arachis hypogaea*	Natural	Galβ1-3GalNAc (T)
33	WFA	*Wisteria floribunda*	*Natural*	Terminal GalNAc, LacDiNAc
34	ACA	*Amaranthus caudatus*	Natural	Galβ1-3GalNAc (T)
35	MPA	*Maclura pomifera*	Natural	Galβ1-3GalNAc (T), GalNAca (Tn)
36	HPA	*Helix pomatia*	*Natural*	αGalNAc (A, Tn)
37	VVA	*Vicia villosa*	*Natural*	α,βGalNAc (A, Tn, LacDiNAc)
38	DBA	*Dolichos biflorus*	Natural	α,βGalNAc (A, Tn, LacDiNAc)
39	SBA	*Glycine max*	Natural	α,βGalNAc (A, Tn, LacDiNAc)
40	Calsepa	*Calystegia sepium*	*Natural*	Biantenna with bisecting GlcNAc
41	PTL I	*Psophocarpus tetragonolobus*	*Natural*	αGalNAc (A, Tn)
42	MAH	*Maackia amurensis*	Natural	α2-3Sia
43	WGA	*Triticum vulgaris*	*Natural*	(GlcNAc)n, polySia
44	GSLIA4	*Griffonia simplicifolia*	Natural	αGalNAc (A, Tn)
45	GSLIB4	*Griffonia simplicifolia*	*Natural*	αGal (B)

[1]Abbreviations: Gal (D-galactose), GalNAc (N-acetyl-galactosamine), GlcNAc (N-acetyl-glucosamine), Fuc (L-fucose), Glc (D-glucose), Sia (Sialic acid), LacNAc (N-acetyl-lactosamine).

[2]Specificity data was obtained by frontal affinity chromatography and glycoconjugate microarray.
